# Miniaturized wireless, skin-integrated sensor networks for quantifying full-body movement behaviors and vital signs in infants

**DOI:** 10.1073/pnas.2104925118

**Published:** 2021-10-18

**Authors:** Hyoyoung Jeong, Sung Soo Kwak, Seokwoo Sohn, Jong Yoon Lee, Young Joong Lee, Megan K. O’Brien, Yoonseok Park, Raudel Avila, Jin-Tae Kim, Jae-Young Yoo, Masahiro Irie, Hokyung Jang, Wei Ouyang, Nicholas Shawen, Youn J. Kang, Seung Sik Kim, Andreas Tzavelis, KunHyuck Lee, Rachel A. Andersen, Yonggang Huang, Arun Jayaraman, Matthew M. Davis, Thomas Shanley, Lauren S. Wakschlag, Sheila Krogh-Jespersen, Shuai Xu, Shirley W. Ryan, Richard L. Lieber, John A. Rogers

**Affiliations:** ^a^Querrey Simpson Institute for Bioelectronics, Northwestern University, Evanston, IL 60208;; ^b^McCormick School of Engineering and Applied Science, Northwestern University, Evanston, IL 60208;; ^c^Sibel Health, Niles, IL 60714;; ^d^Department of Mechanical Engineering, Northwestern University, Evanston, IL 60208;; ^e^Max Nader Center for Rehabilitation Technologies and Outcomes Research, Shirley Ryan AbilityLab, Chicago, IL 60611;; ^f^Department of Physical Medicine & Rehabilitation, Feinberg School of Medicine, Northwestern University, Chicago, IL 60611;; ^g^Department of Electrical and Computer Engineering, University of Wisconsin–Madison, Madison, WI 53706;; ^h^Department of Biomedical Engineering, Northwestern University, Evanston, IL 60208;; ^i^Medical Scientist Training Program, Feinberg School of Medicine, Northwestern University, Chicago, IL 60611;; ^j^Department of Materials Science Engineering, Northwestern University, Evanston, IL 60208;; ^k^Department of Civil and Environmental Engineering, Northwestern University, Evanston, IL 60208;; ^l^Department of Physical Therapy & Human Movement Sciences, Feinberg School of Medicine, Northwestern University, Chicago, IL 60611;; ^m^Department of Microbiology–Immunology, Feinberg School of Medicine, Northwestern University, Chicago, IL 60611;; ^n^Department of Pediatrics, Ann & Robert H. Lurie Children’s Hospital of Chicago, Feinberg School of Medicine, Northwestern University, Chicago, IL 60611;; ^o^Department of Medical Social Sciences, Feinberg School of Medicine, Northwestern University, Chicago, IL 60611;; ^p^Institute for Innovations in Developmental Sciences, Northwestern University, Chicago, IL 60611;; ^q^Department of Dermatology, Feinberg School of Medicine, Northwestern University, Chicago, IL 60611;; ^r^Shirley Ryan AbilityLab, Chicago, IL 60611;; ^s^Edward R. Hines Jr. VA Medical Center, Maywood, IL 60141;; ^t^Department of Electrical and Computer Engineering, Northwestern University, Evanston, IL 60208;; ^u^Department of Chemistry, Northwestern University, Evanston, IL 60208;; ^v^Department of Neurological Surgery, Northwestern University, Evanston, IL 60611

**Keywords:** wireless sensor networks, movement behaviors, infants motor skill, motion recapitulation

## Abstract

Early identification of atypical infant movement behaviors consistent with underlying neuromotor pathologies can expedite timely enrollment in therapeutic interventions that exploit inherent neuroplasticity to promote recovery. Traditional neuromotor assessments rely on qualitative evaluations performed by specially trained personnel, mostly available in tertiary medical centers or specialized facilities. Such approaches are high in cost, require geographic proximity to advanced healthcare resources, and yield mostly qualitative insight. This paper introduces a simple, low-cost alternative in the form of a technology customized for quantitatively capturing continuous, full-body kinematics of infants during free living conditions at home or in clinical settings while simultaneously recording essential vital signs data. The system consists of a wireless network of small, flexible inertial sensors placed at strategic locations across the body and operated in a wide-bandwidth and time-synchronized fashion. The data serve as the basis for reconstructing three-dimensional motions in avatar form without the need for video recordings and associated privacy concerns, for remote visual assessments by experts. These quantitative measurements can also be presented in graphical format and analyzed with machine-learning techniques, with potential to automate and systematize traditional motor assessments. Clinical implementations with infants at low and at elevated risks for atypical neuromotor development illustrates application of this system in quantitative and semiquantitative assessments of patterns of gross motor skills, along with body temperature, heart rate, and respiratory rate, from long-term and follow-up measurements over a 3-mo period following birth. The engineering aspects are compatible for scaled deployment, with the potential to improve health outcomes for children worldwide via early, pragmatic detection methods.

## Significance

Early detection of infant neuromotor pathologies is critical for timely therapeutic interventions that rely on early-life neuroplasticity. Traditional assessments rely on subjective expert evaluations or specialized medical facilities, making them challenging to scale in remote and/or resource-constrained settings. The results presented here aim to democratize these evaluations using wireless networks of miniaturized, skin-integrated sensors that digitize movement behaviors and vital signs of infants in a cost-effective manner. The resulting data yield full-body motion reconstructions in the form of deidentified infant avatars, along with a range of important cardiopulmonary information. This technology approach enables rapid, routine evaluations of infants at any age via an engineering platform that has potential for use in nearly any setting across developed and developing countries alike.

A growing body of literature suggests that infant movement behaviors can reveal rich information about the developing central nervous system. Atypical patterns can indicate underlying neuromotor pathologies that reflect or presage clinical conditions like cerebral palsy, autism spectrum disorder, or minor forms of other neurological dysfunctions (1–4). Detection at the earliest time in infancy is critically important for initiating clinical interventions that harness inherent neuroplasticity to promote early recovery and optimal long-term functional outcomes and quality of life (5–7). Methods that enable detection even prior to conventional clinical identification are of particular interest.

Currently, diagnoses of infant neuromotor disorders rely on a combination of medical history records, clinical examinations, and imaging methods. Diagnostic screening for atypical development varies by age and risk factors, wherein children with prebirth, during-birth, and postbirth medical complications or obviously observed developmental delays receive enhanced, in-depth clinical testing (8, 9). Recent international protocols recommend additional evaluations for at-risk infants early in life, including visual observations of the infant’s general movements (GM) by an expert clinician throughout the preterm and term phase (2, 4, 10). Though assessment of GM has value in screening of certain neuromotor pathologies, this approach is subjective, labor intensive, and requires specialized training, which may hinder its scalability to remote geographical locations or to developing countries.

Alternative strategies for high-resolution analysis of an infant’s motor development involve quantitative data from digital imaging systems or commercial wearable sensors packages (11–15), often also with electroencephalography (16) or electromyography (17). A key drawback is that these approaches rely on cumbersome and expensive hardware, suitable for use only in tertiary medical centers or specialized facilities. A comparison of systems that support motion analysis appears in *SI Appendix*, Table S1 with detailed features and specifications associated with device size, weight, mechanics, and costs. Consumer-friendly imaging platforms, such as the now-discontinued Microsoft Kinect (18) and related systems, can be used to track body movements, but privacy concerns may limit their real-world implementation in community clinics or home settings. Such approaches may also lack the necessary precision and resolution to accurately track natural motions of neonates and young children, whose body configurations differ from adults and are often not represented substantially in training data for pose estimation algorithms (19). None of these various systems for motion analysis provides capabilities in vital signs monitoring. An urgent unmet need is, therefore, in affordable, adaptable, high-resolution systems to measure and evaluate movements and key physiological health metrics in infants across the risk spectrum. Such technologies may enable detection and quantification of atypical motor development earlier and more precisely compared to that possible with current clinical screening tools. Importantly, technologies that record and analyze data continuously without the need for trained personnel can be deployed to a variety of settings, either inside or outside of the clinic, to better capture the range of natural patterns of motor behavior that may occur in daily life in a scalable manner. Improved detection could then lead to enhanced interventions at the earliest stages of life, across a broad spectrum of conditions and circumstances, thus limiting disability while enhancing recovery.

To address these needs, this paper introduces an easy-to-use, low-cost, flexible, wireless sensor platform designed specifically for quantitatively measuring patterns of movements and vital signs characteristics in infants. The wireless network platform includes a collection of miniaturized, soft devices, which we refer to as Core Optimization for Regulation of Babies (CORB) sensors, each of which operates in a time-coordinated fashion to record data from three-axis digital accelerometers and gyroscopes. Flexible CORB sensors are more than three times thinner, five times lighter, and two times smaller in overall volume (*SI Appendix*, Table S1) than the most advanced commercialized sensors for motion capture (Xsens MTw Awinda, Xsens Technologies B.V.), and they also uniquely enable precise measurements of cardiopulmonary function and body temperature. Due to their lightweight construction and miniaturized form factor, CORB sensors can be applied with readily available medical silicone adhesives (2477P, 3M) or with bands or wraps. When placed at strategic locations on the body of an infant or child, CORB sensors capture gross and subtle movements across the full range of spatiotemporal scales. The results provide direct, quantitative assessments of body dynamics and can be used to reconstruct the continuous movement of the arms, legs, torso, and head in avatar form. High-bandwidth units placed on the chest simultaneously capture additional data related to standard and nonstandard physiological markers of health status (i.e., heart rate, heart rate variability, respiratory rate and sounds, body temperature, and patterns of vocalization). The following describes all aspects of device design and operation, with proof-of-concept testing on infants with low and elevated risk profiles, each at term-corrected ages of 1 wk, 1 mo, and 3 mo.

## Results and Discussion

### Miniaturized Wireless Devices for Infants.

**Fig. 1.**
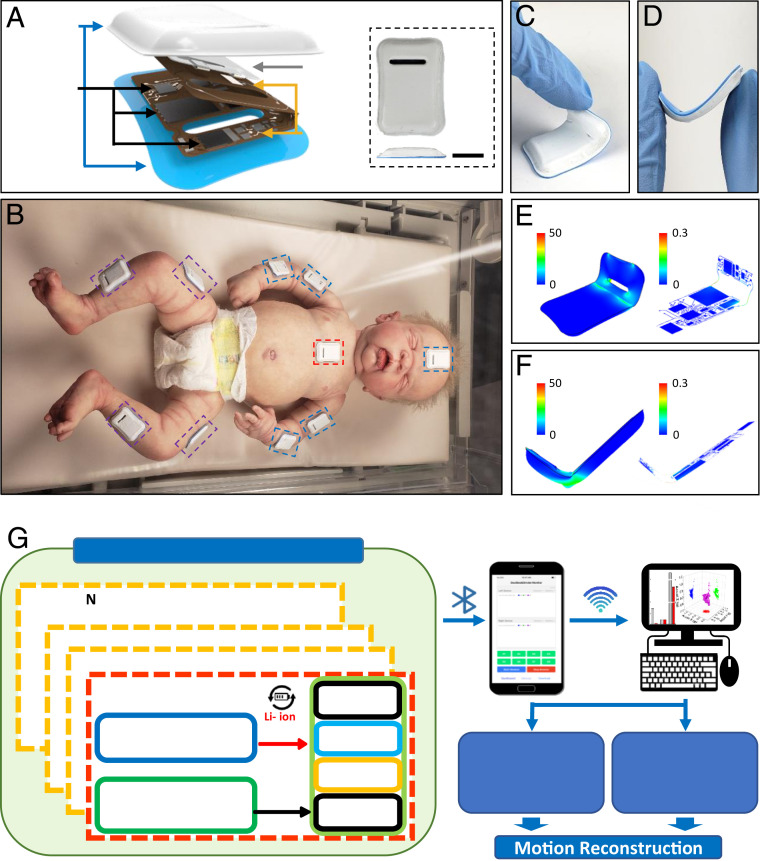
Images, schematic illustrations, and functional flow charts for miniaturized wireless sensors (i.e., CORB sensors) designed for quantifying gross motor behaviors and vital signs in infants. (*A*) Exploded-view schematic illustration of the device. Optical image of the device (*Inset*). (Scale bar, 1 cm.) (*B*) Measurement configuration for capturing full body motions, illustrated with a baby doll. (*C–F*) Images and finite element analysis computations of a device flexibility during various mechanical deformations: peeling (*C* and *E*) and bending with an angle of 90° (*D* and *F*). (*G*) Functional diagram of the platform showing hardware blocks including the power management, Bluetooth radio, microcontroller, memory, and six-axis inertial measurement unit for each device. The collected data from each device include synchronized timestamps to ensure millisecond relative timing accuracy. A user interface on a smartphone or table controls the devices, captures real-time data, and supports data downloads a 3D motion reconstruction using a local PC.

The thin, miniaturized geometries, soft mechanical properties, and wireless operational capabilities of CORB sensors facilitate gentle bonding to the sensitive skin of infants, with minimal mass or mechanical load. Continuous, time-synchronized sensing from multiple locations yields quantitative, precise data on body motions. In a representative use case, these sensors measure three-axis accelerations and angular velocities from devices mounted on the chest, the forehead, and strategic positions across the limbs (Fig. 1) to capture essential characteristics of full-body motions. The schematic illustration in Fig. 1*A* shows the overall size (32 × 21 × 3 mm) and shape of a device, each of which weighs less than 2.6 g. A thin silicone elastomer forms a water-tight enclosure (0.3 mm thick, Silbione RTV 4420) that also mechanically isolates the active and passive components to ensure a gentle interface to the skin. A 10 × 1.5-mm hole formed through the functional and encapsulation layers along one side not only enhances the mechanical flexibility of the devices while mounted on the skin but also provides a highly bendable location from which to initiate peeling during removal. Fig. 1*B* shows 10 such devices mounted on a model infant in a layout designed for full-body motion assessments per protocols of the GM, spanning the middle of the upper and lower arms, middle of the thighs and shins, chest, and forehead. Fig. 1 *C*–*F* show the device bent and twisted in various ways, including those associated with peeling (Fig. 1 *C* and *E*) and bending at an angle of 45° (*SI Appendix*, Fig. S1) to 90° (Fig. 1 *D* and *F*). Finite element analysis confirms that the strains in the copper (Cu) traces of this system remain below the yield limits (ε = 0.3%) for bending radii as small as ∼2.8 mm (Fig. 1 *E* and *F* and *SI Appendix*, Fig. S1). The miniaturized dimensions of the devices and their compliant mechanical properties facilitate mounting on small and highly curved parts of the body with minimal stresses on the skin during application, use, and removal. These features are especially important for the vulnerable patient populations considered here.

The functional diagram in Fig. 1*G* summarizes the overall architecture and data flow. The system includes three main components: a time-synchronized collection of CORB sensors, a user interface based on a customized app that operates on a smartphone or tablet, and a set of algorithms for motion reconstruction, implemented on a local personal computer (PC). A primary sensor communicates with the other sensors to synchronize the local time as essential information for reconstructing full body motions without drift, latency, or discordance. This synchronization strategy exploits a custom 2.4-GHz wireless transmission scheme in a star topology, where each node has a 32.768-kHz clock timer to timestamp the data. The primary device broadcasts its local time once per second to the other sensors. Each of these sensors then synchronizes its local time accordingly. Experimental data shown subsequently indicate that submillisecond time accuracy can be achieved across all of the sensors, without any long-term drift. Under the control of an interrogator via Bluetooth Low Energy (BLE), each sensor continuously collects responses from a three-axis accelerometer and a three-axis gyroscope and wirelessly transmits data with these synchronized timestamps in real time to the user interface. The sensors also simultaneously store these data onto an internal memory module. Passing the data to a local PC via BLE protocols allows processing to yield movement information from each sensor in the network, as well as static and dynamic orientations relative to the gravity vector, thereby allowing for reconstruction of full-body motions. Data from the chest sensor can also be analyzed to yield cardiopulmonary and vocalization sounds, along with heart and respiratory rate and their cycle-to-cycle variability, and an estimate of core body temperature.

**Fig. 2.**
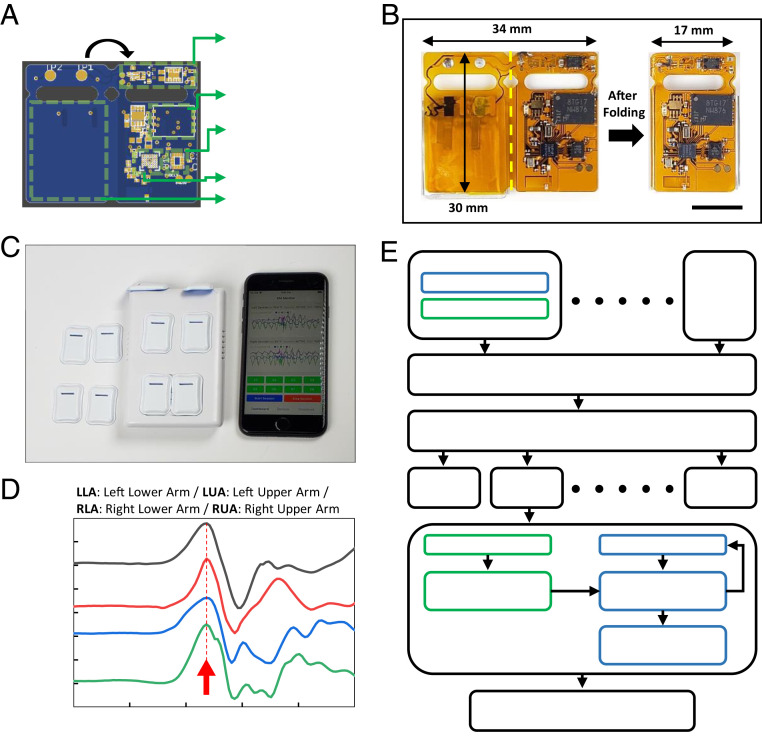
Schematic illustrations and optical images of the sensor configuration and data flows for 3D motion reconstruction. (*A*) Schematic illustration of the flexible PCB that supports the power management and battery protection SoC, 4Gb NAND flash memory, six-axis inertial measurement unit, Bluetooth low energy SoC, and lithium-polymer battery. (*B*) Optical image of the fPCB after mounting all components (*Left*) and after folding (*Right*). (Scale bar, 1 cm.) (*C*) Image of the full system, including multiple time-synchronized devices, a charger, and user interface on a smartphone. (*D*) Time-synchronized and normalized accelerometer signals acquired from four sensors on the left upper arm (LUA), left lower arm (LLA), right upper arm (RUA), and right lower arm (RLA). The red arrow indicates the timing of a jumping movement. The results indicate time synchronized operation to within 10 ms or less. (*E*) Algorithm flowchart for motion reconstruction. The *Top* frames highlight the steps in bias correction, signal filtering, and time synchronization for each device. The *Middle* frames show static attitude extraction from the three-axis accelerometer and dynamic attitude extraction from the three-axis gyroscope. The *Bottom* frame corresponds to transfer of resulting data to the ROS for 3D motion reconstruction.

Fig. 2*A* shows the layout of a flexible printed circuit board (fPCB) platform that supports all of the electronic components in an overall layout designed to fold in half to yield the final form of the device. The key components include passive elements with 0603 and 1005 (metric code) mm footprints, a voltage and current protection integrated circuit (BQ2970, Texas Instruments), a 3.0-V step-down buck DCDC converter (TPS62740, Texas Instruments), a 4-Gb nonvolatile NAND flash memory (MT29F4G, Micron), a low-power inertial measurement unit (IMU) that includes an accelerometer and gyroscope (BMI160, BOSCH), BLE system on a chip (SoC) (nRF52832, Nordic Semiconductor), and a 3.7-V lithium polymer battery (7 mAh, 0.9 mm thick; PowerStream) which can support more than 5 h of continuous operation with real-time data streaming and data-saving capability (*SI Appendix*, Fig. S2). The dimensions of the fPCB are 34 × 30 mm and 17 × 30 mm before and after folding, respectively, at a location defined by the yellow dashed line (Fig. 2*B*). Thin top and bottom layers of a silicone elastomer (0.3 mm thick) formed using a pair of concave and convex aluminum molds enclose the device except at two small circular openings (2.0-mm diameter) to electrode pads for charging the battery. The process for encapsulation involves placing the folded fPCB between these layers of elastomer and filling the remaining spaces with a low-modulus silicone material (Eco-Flex 00-30). This material acts as a soft, conformal buffer around the rigid electronic components, and it provides mechanical isolation during manipulations involved in applying and removing the devices from the skin.

Fig. 2*C* shows a collection of sensors and a user interface on a smartphone. Fig. 2*D* presents *y*-axis accelerometer data from four sensors worn on the left (upper and lower) and right (upper and lower) arm that capture a jumping motion executed by the subject. The position of the peak associated with the jump (red arrow in Fig. 2*D*) reveals that the time difference among the sensors is less than 1 ms, which is negligible for applications considered here. When interpreted using appropriate algorithms, the raw data collected in this manner can be directly relevant to assessing motion disorders without further manipulations. In many cases, however, visual inspection of full body motions can also be useful. The following sections highlight approaches for reconstructing such motions in avatar form from the sensor data.

### Approaches to Motion Reconstruction.

The algorithm for reconstruction uses measurements of linear acceleration and angular velocity, each along three orthogonal axes, to reproduce points across a three-dimensional (3D) humanoid model based on the Robot Operating System (ROS) platform, with the Rviz 3D visualization tool. The results quantify gross motion levels as well as behaviors relevant to GM assessments and diagnosis of atypical motor development patterns such as those associated with cerebral palsy. The block diagram in Fig. 2*E* illustrates the overall flow, from data collection to motion reconstruction. The process of estimating pose and orientation of a sensor by analyzing the linear acceleration and angular velocity data is known as dead reckoning. Estimating the orientation of each sensor at each time step based on a measurement of angular velocity at that time step and sequential Euler rotation around each axis can introduce systematic error, as the rotation is noncommutative. Simultaneous computations of the rotation minimize these errors (20), as executed in software code written in Python 2.7.15+ and ROS Melodic, available on a web-based source code management cloud (21). The three main function scripts are for data transformation, data parsing, and computation. The transformation script captures the position and the 3D rotation group (so3 matrix) components and then broadcasts the results to the Transforms ROS package in quaternion angle. The parser script imports data from the sensor measurements in raw format and parses them into individual containers of three-axis acceleration and three-axis angular velocity. The computation script computes the position and orientation of the sensor frame of reference in a 3D environment and saves the results in a specific file format to broadcast to the ROS platform. This software addresses the main technical challenges, as summarized in *A Simple Alternative to Dead Reckoning*.

### A Simple Alternative to Dead Reckoning.

In dead reckoning, double integration of the linear acceleration to yield 3D positions leads to an unacceptable accumulation of small calibration errors, sources of noise, and other effects. The additional use of data from magnetometers and/or global positioning system components can reduce these errors, but their integration into the device platform greatly increases power consumption and overall size and weight. A simple alternative exploits constraints associated with a body model, using the unified robot description format. This scheme relies only on the orientations, as determined by integration of the angular velocity.

### Methods for Reducing Sensor Bias and Filtering the Data.

All sensors carry some degree of bias and noise. Both effects can be observed from measurements obtained from sensors at rest *SI Appendix*, Fig. S3. The bias appears as the distance of the solid lines for each axis of measurement from absolute zero in the *y*-axis. Noise corresponds to high-frequency temporal fluctuations (*SI Appendix*, Fig. S3). Although bias can be manually balanced by elementwise subtraction of individual bias values from measurement data, it can also be minimized through sensor calibration at the firmware level. In this approach, compensation occurs each time a sensor wirelessly connects, as performed in the firmware inline. Compensation values stored in the nonvolatile memory can be loaded into the registers and utilized on prefiltered data. *SI Appendix*, Fig. S4 shows plots of the spectral characteristics of a single stationary sensor, where the bias appears at 0 Hz (*SI Appendix*, Fig. S4*A*), and of a single sensor mounted on the arm of a human subject (*SI Appendix*, Fig. S4*B*). Consistent with the published literature, the relevant frequency range for human motion is between 0 and 20 Hz. A digital low-pass filter (fifth-order Butterworth) integrated in the data-parsing script removes features with frequencies higher than 20 Hz. The frequency response of this filter appears in *SI Appendix*, Fig. S5*A*. The plot in *SI Appendix*, Fig. S5*B* shows the signal after filtering. Related filtering schemes can be applied to data from the chest sensor to isolate vital signs information and vocalization events as described subsequently.

### Reconstructing Full-Body 3D Motions from Sensor Data.

**Fig. 3.**
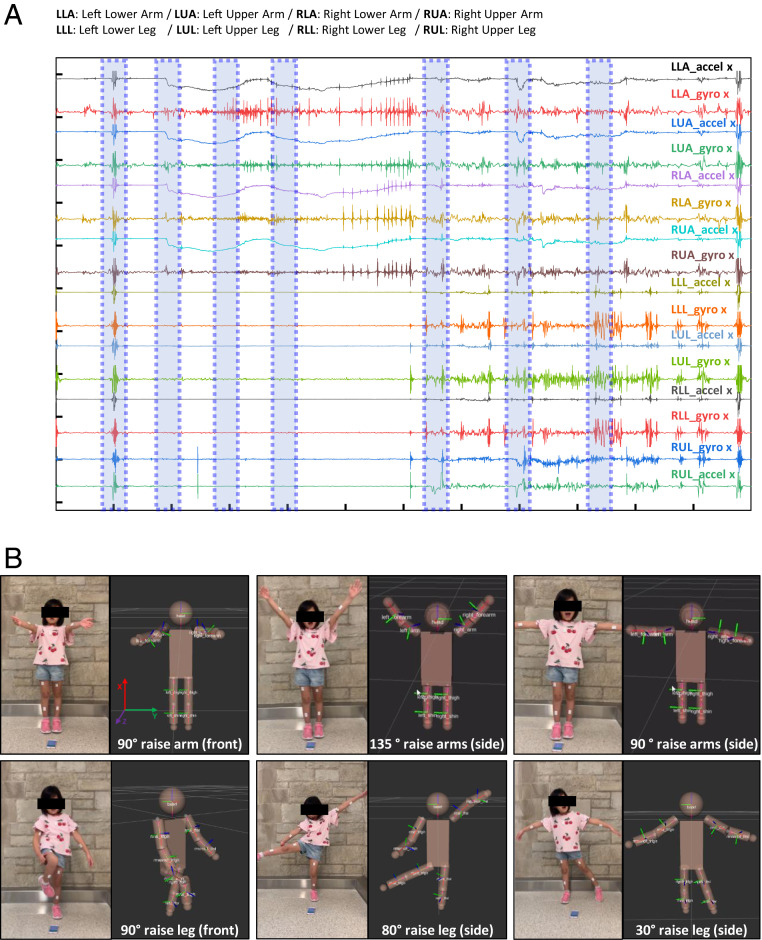
Representative data collected from a child subject during various arm and leg motions with different angles and orientations. (*A*) Normalized acceleration and gyroscope data during motion. The subject jumped (around 10 s), raised left and right arms to the front at 90° (around 19 s), and to the side at 135° (around 29 s) and 90° (around 39 s). (*B*) Optical and 3D reconstructed images from these motion data. 1: raised left and right arms to the front at 90°; 2: raised left and right arms to the side at 135°; 3: raised left and right arms to the side at 90°; 4: raised left arms to the side at 30°and right leg to the front at 90°; 5: raised left and right arms to the side at 45°and 120 °, respectively, and right leg to the side at 80°; and 6: raised left and right arms to the side at 90°and left leg to the side at 30°.

As mentioned previously, full-body motion sensing requires time-synchronized operation of a collection of sensors mounted at strategic locations. Examples presented here use nine sensors—a primary sensor in the middle of the body (chest) and secondary sensors on the limbs (two on each). Careful selection of the wireless transmitting power and the matching conditions for the antenna of each device ensures robust, power-efficient operation in simultaneous streaming mode as an alternative to a mode that involves local storage of data. Fig. 3 summarizes results from sensors that record data from a three-axis accelerometer (16 bit, 16,384 LSB/g sensitivity for gravitational acceleration) at a high sampling rate (200 Hz, adjustable up to 1,600 Hz) and a wide dynamic range (±8 g, adjustable up to ± 16 g) and from a three-axis gyroscope (262.4 LSB/°/s sensitivity for angular velocity) also at high sampling rate (200 Hz, adjustable up to 3,200 Hz) and a wide dynamic range (1,000°/s, adjustable up to 2,000°/s). These parameters are sufficient for accurate recordings of motions of children and infants (12).

Fig. 3 illustrates an example of a healthy child (7 y old, 118 cm tall) with CORB sensors placed on the right upper arm (RUA), right lower arm (RLA), left upper arm (LUA), left lower arm (LLA), right upper leg (RUL, thigh), right lower leg (RLL, shin), left upper leg (RUL, thigh), left lower leg (RLL, shin), and chest. In the initial configuration, all sensors face forward, with the subject in a normal standing position (*SI Appendix*, Fig. S6) and placed on the middle of the arm/leg to collect the most accurate motion angles and accelerations for reconstruction processing. Fig. 3*A* shows representative static and dynamic acceleration and angular velocity data collected during different controlled motions (*SI Appendix*, Fig. S7), as described in Fig. 3*B* with matching optical images and corresponding 3D reconstruction results. In particular, 1) raised left and right arms to the front at 90°, 2) raised left and right arms to the side at 135°, 3) raised left and right arms to the side at 90°, 4) raised left arms to the side at 30° and right leg to the front at 90°, 5) raised left and right arms to the side at 45 and 120°, respectively, and right leg to the side at 80°, and 6) raised left and right arms to the side at 90° and left leg to the side at 30°. Detailed information appears in *SI Appendix*, Figs. S8–S10 and Movies S1–S4 for different motions, including quantitative comparisons between video processing and reconstruction results measured from a human arm mimic model (shoulder-upper-lower arm). Controlled upper and lower arm motions measured for 100 s (30 times repetitive motion, ∼90° change from the starting position) (*SI Appendix*, Fig. S9*A*) yield trajectory angle profiles from reconstructions based on video recordings and CORB sensor data, as shown in *SI Appendix*, Fig. S9 *B*–*D*. The results indicate that the differences between these two methods are less than 0.2° for the initial configuration and that they increase over the time of the measurement to a value of 1.3° (*SI Appendix*, Fig. S10). The drift (0.013°/s) accumulates over the course of a single session but is reset during the initialization step of ROS.

Synchronized merged videos of motion tracking and results of CORB sensor reconstruction are in Movie S1. Movie S2 summarizes data collected with two sensors on each arm, one on the forearm and the other on the upper arm. The results show that reconstruction accurately captures the orientations and angles of the subject’s arms. A demonstration of data collected with eight sensors attached to the body is in Movies S3 and S4. Here, the subject has one sensor on each of the limbs, both sides of the upper arm, forearm, thigh, and shin while walking. The platform yields full 360° 3D movements (*SI Appendix*, Fig. S11) with quantitative information.

The method for reconstruction does, however, demand some knowledge of the initial body position as well as lengths of the limbs, legs, and core body for an accurate personalized 3D full-body reconstruction model. Indirect sensing (e.g., multicamera) alternatives bypass these requirements, but they typically require complex, costly setups that can be applied only to a single well-defined space, without the ability to track movements during natural daily activities. Privacy issues represent additional concerns for many parents. As mentioned before, direct sensing schemes (e.g., inertial sensors, magnetic tracking devices) are not optimally suited for infants due to bulk, size, rigid construction, and/or wired connections (22).

### Quantifying Gross Motor Behaviors in Infants.

Results presented here focus on feasibility demonstrations of CORB sensor networks on two infants, one assessed at low risk (LR) of atypical motor development and one at elevated risk (ER) based on various factors (birth at 34 wk gestational age, low birthweight, multiple birth, and treatment for 2 wk in a neonatal intensive care unit). A total of 10 CORB sensors placed bilaterally at the shoulder, wrist, hip, and ankle, the xiphoid process, and forehead capture movements continuously and in real time (*SI Appendix*, Fig. S12). The infant’s movements include various postures (i.e., prone, supine, supported sitting, supported standing, and horizontal suspension) performed at ages of ∼1 wk, 1 mo, and 3 mo. For the 1-wk and 1-mo cases, only six devices were used—at the wrists, ankles, chest, and forehead—limited by the small sizes of the infants at these ages. Normalized three-axis accelerometer and three-axis gyroscope signals from LR and ER infants at 1 wk (*SI Appendix*, Fig. S13), 1 mo (*SI Appendix*, Fig. S14), and 3 mo old (*SI Appendix*, Fig. S15) appear in *SI Appendix*, Figs. S3–S15.

**Fig. 4.**
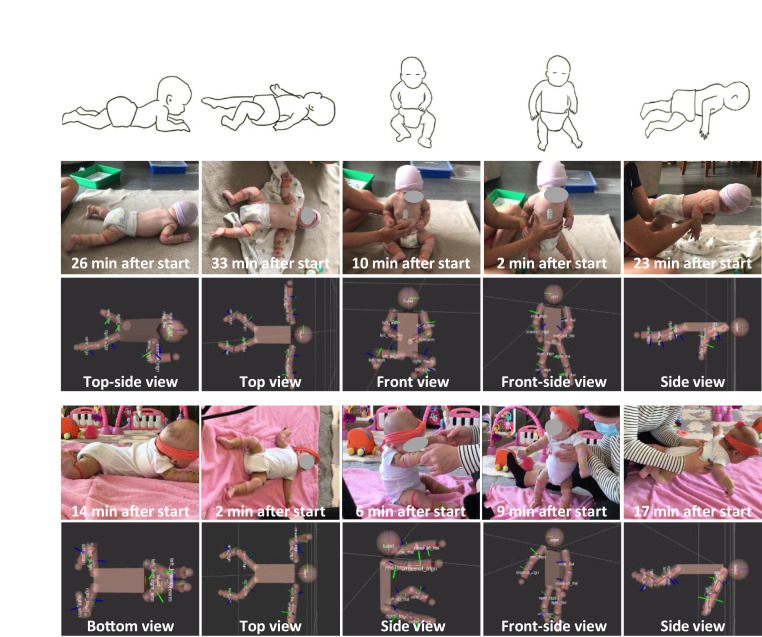
Comparisons of representative gross motor behaviors associated with large physical motions from 3-mo-old infants at LR and ER of atypical motor development. LR and ER infants’ motor behaviors and corresponding optical images captured over the course of the 35-min session and associated 3D motion reconstruction results with different viewpoints. Captured time points for pictures and reconstruction results are indicated at the *Bottom* of each picture.

**Fig. 5.**
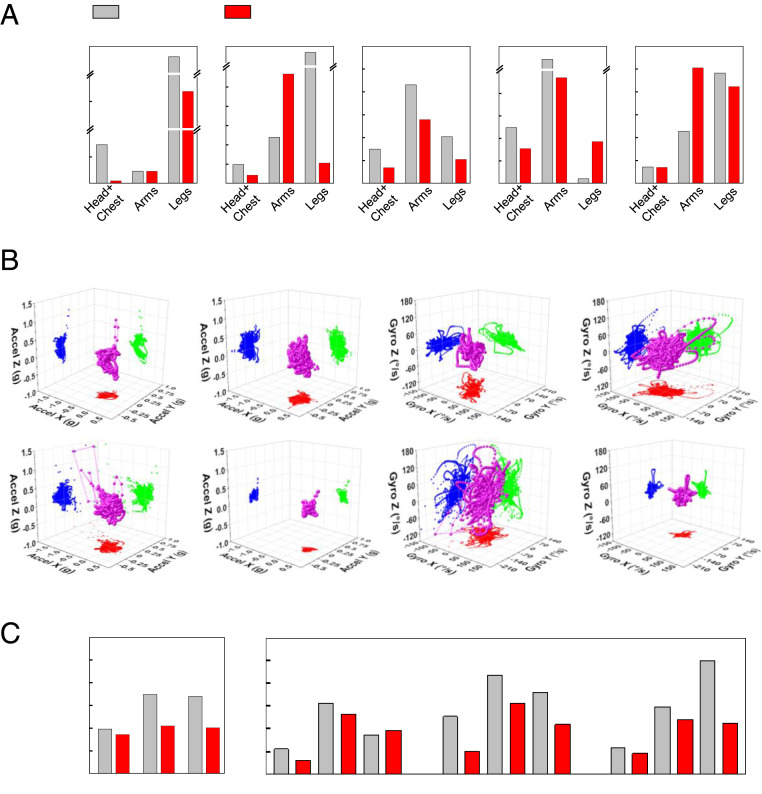
Quantitative comparisons of long-term and follow-up measurement results over a 3-mo period from representative infants at LR and ER of atypical motor development. (*A*) QAL comparisons of 3-mo-old LR and ER subjects in various postures such as prone, supine, supported sitting, supported standing, and horizontal suspension corresponding to the images in Fig 4. (*B*) 3D scatter plots from 3-mo-old LR and ER subjects‘ three-axis inertial measurements in supine position (magenta sphere). Acceleration of right and left arms from LR (first and second columns on the *Top* row) and ER subjects (first and second columns on the *Bottom* row). Angular velocity of right and left arms from LR (third and fourth columns on the *Top* row) and ER subjects (first and second columns on the *Bottom* row). XY (red), YZ (blue), and ZX (green) projections on each plot. (*C*) Overall QAL (*Left*) and each level from head and chest/arms/legs (*Right*) from the LR and ER infants when subjects are 1 wk, 1 mo, and 3 mo old.

Figs. 4 and 5 summarize results for representative LR (subject ID: LR_1) and ER (subject ID: ER_1) infants at 3 mo of age (23). Visual observations and video analysis (Fig. 4 and Movie S5) do not reveal obvious gross motor deficiencies of the ER infant such as difficulty in lifting the head or holding the head up while in prone position/standing/sitting/in horizontal suspension, or in exhibiting stiffness in the legs/arms with little or no movements. Such behaviors at this age could be indicative of delayed or atypical neuromotor development (23). Both infants demonstrate variability in their whole-body postures, as captured in the motion reconstructed from the sensor data. Unlike video analysis, 3D reconstruction enables quantitative analysis from any viewing angle, including those that might be concealed in the video analysis. For example, video analysis of the ER subject in the prone posture does not allow for assessments of the symmetry of motions and orientations of both arms and legs. Fig. 5 highlights values for quantitative activity level (QAL) calculated by combining the spectral power from 0.1 to 10 Hz (24–27) of each sensor and integrating over equal time for both subjects. The details of the computational approach are in *SI Appendix*, Fig. S16. Comparisons of this metric for 3-mo-old LR and ER subjects in prone, supine, supported sitting, supported standing, and horizontal suspension positions appear in Fig. 5*A*. These initial feasibility data indicate that in most cases examined in these brief episodes, with the exception of arms in the supine position, legs in the supported standing position, and arms in the horizontal suspension position, the QAL values for the LR infant are higher than those of the ER infant. Specific values are in *SI Appendix*, Table S2.

The raw data can be displayed in different graphical forms for further visual analysis. An example of 3D scatter plots of accelerations measured from 3-mo-old LR and ER subjects in the supine position (magenta sphere) appear in Fig. 5*B* for rapid assessments of movement behavior characteristics with respect to their symmetry and variability. The first and second and the third and fourth columns show distributions of the acceleration and angular velocity, respectively, for the right and left arms of the LR (*Top*) and ER subjects (*Bottom*). These representations clearly show that the movement behavior of the ER infant is more sporadic and less symmetrical than that of the LR infant.

Overall QAL values (Fig. 5 *C*, Left) reveal differences between the LR (subject ID: LR_1) and ER (subject ID: ER_1) subjects at three timepoints after birth (Fig. 5 *C*, Right). Specifically, the QAL values are 14, 66, and 68% higher in the LR infant than those in ER infant at 1 wk, 1 mo, and 3 mo old, respectively. Averaged values for each sensor (head + chest, arms, and legs) provide additional insights, consistent with qualitative evaluations described in Fig. 4. For instance, at 1 mo, the QAL of the head and chest of the LR infant is 154% greater than that of the ER infant. In a similar way, the QAL of the arms and legs are 40 and 64% higher for the LR than the ER infant. Also at 3 mo, the QAL of the combined head and chest movements of the LR infant is 27% higher in LR than in ER infants. In a similar way, QAL for the arms and legs are 23 and 124% higher, respectively, than in ER infants. Specific values are in *SI Appendix*, Table S3. The various differences described here may correspond to intersubject movement variability rather than underlying neuromotor disorders. Preliminary results from 10 infants (6 LR and 4 ER subjects) suggest differences in QAL between LR and ER subjects, particularly in movement of the arms and legs (*SI Appendix*, Figs. S17 and S18 and Table S4). Additional testing and larger-scale data analysis from children with known typical and atypical development trajectories will define normative ranges of sensor-based motion metrics throughout early infancy and childhood.

A final note is that the sensors have utility not only in assessments but also in tracking of physical therapies that involve controlled placement of the infant into different body positions, in one example sometimes known as Tummy Time. For example, *SI Appendix*, Fig. S19 shows the body orientation extracted from the chest sensor data of the LR and ER subjects. The results can be used to determine the cumulative time of each posture including prone and horizontal suspension (body angle > 135°, red shaded region in *SI Appendix*, Fig. S19), supine (body angle < 25°), and sitting and standing (25° < body angle > 135°). These values, here not associated with a particular therapy, are within the range expected for healthy resting newborns and infants (28, 29).

### Capturing Vital Signs Information in Infants.

**Fig. 6.**
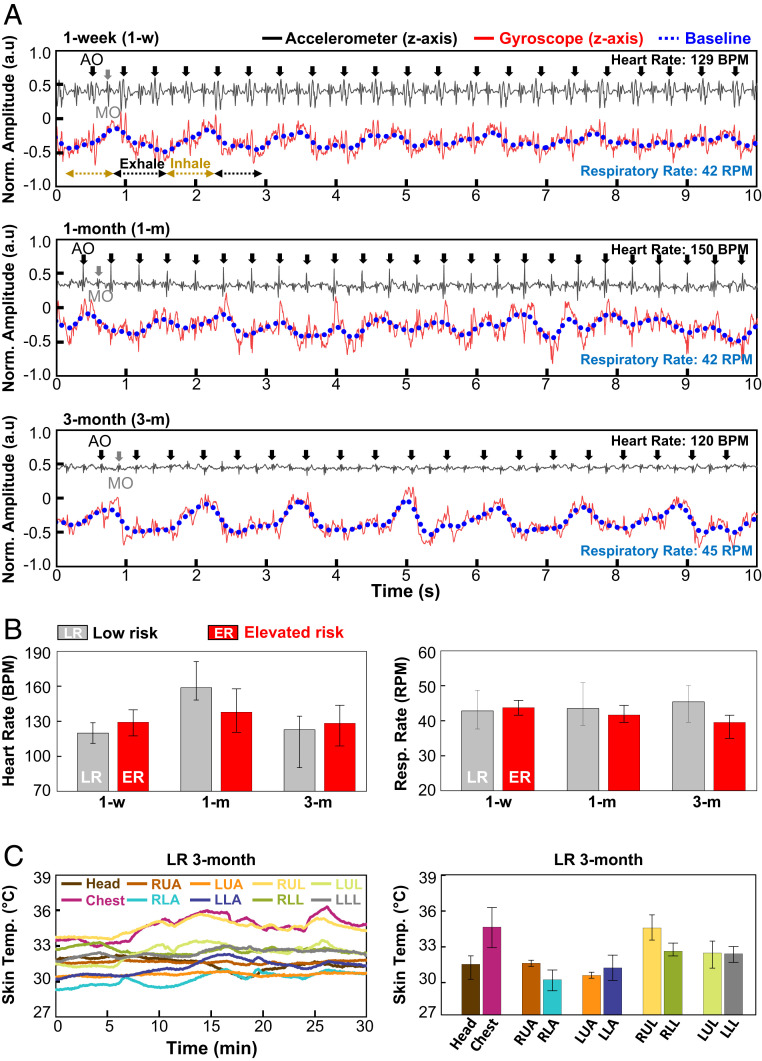
Quantitative long-term and follow-up cardiac and respiratory activity measurement results over a 3-mo period from infants at LR and ER. (*A*) Normalized chest sensor signal from LR infants at 1 wk (*Top*), 1 mo (*Middle*), and 3 mo of age (*Bottom*). The black line corresponds to data from the *z*-axis of the accelerometer (seismocardiogram); the red line is the *z*-axis gyroscope signal; and the blue dashed line is a running average of this gyroscope signal (respiratory activity). AO and MO denote aortic and mitral valve opening, respectively. (*B*) Heart rate (*Left*) and respiratory rate (*Right*) determined from these data, LR (gray filling bar) and ER (red filling bar) subjects including error bars. (*C*) Time-synchronized full-body temperature monitoring from 3-mo-old LR infants collected by the CORB sensors. Temperature variation during data collection over 30 min (*Left*). Skin temperature determined from each device on the head, chest, and limbs including error bars (*Right*).

As mentioned previously, the high-frequency information in data captured using these same CORB sensors contain vital signs and other physiological information associated with mechanoacoustic signatures of internal body processes, highlighting the ability to capture heart rate and respiratory rate from the sensor on the chest (*SI Appendix*, Fig. S16 *B* and *C*). Fig. 6*A* shows the normalized *z*-axis accelerometer (black solid), *z*-axis gyroscope (red solid) signals, and the extracted baseline of the gyroscope signal (blue dot). For example, the *z*-axis accelerometer data from the chest sensor of a 1-wk-old subject (*Top* in Fig. 6*A*) contains clear information on cardiac activity, as a seismocardiogram with prominent peaks induced by Aortic valve opening (black arrow) and Mitral valve opening (gray arrow) (30–32). Using a relatively low sampling frequency setting (200 Hz) for power optimization prevents collection of phonocardiogram signal, such as heart murmurs. However, IMUs with high sampling frequency characteristics (3.3 or 6.6 kHz) can capture high spectral features to measure acoustic signals in addition to mechanical signals induced by cardiac activities (26, 27). The baseline of the gyroscope signal (blue dashed line) features a clear and periodic response associated with exhalation (black dashed line) and inspiration (brown dashed line). Comparisons of results from the CORB sensor and those from a Food and Drug Administration (FDA)–approved capnography device (EMMA, Masimo) recorded from three adult subject volunteers show excellent agreement in respiratory rate, as presented in *SI Appendix*, Fig. S20. Bland–Altman plots of the two methods are in *SI Appendix*, Fig. S21. Measurements with the CORB sensor (three subjects, 1,421 data points) yield a mean difference of 0.01 respirations per minute (RPM) and a SD of 0.53 RPM compared to the FDA-approved system. Comparison data were not collected from infants due to size constraints with the capnography device used here. Data collected in follow-up measurements from 1- and 3-mo-old subjects also shows noticeable responses but with different signal amplitudes compared to those from the 1-wk-old subject (Fig. 6*A*). Specifically, the amplitude of the *z*-axis acceleration is 50% higher for the 1-wk-old subject compared to the value in the 3-mo-old subject. Likewise, the fluctuations of the gyroscope signal corresponding to respirations are clearer and stronger for the 3-mo-old subject compared to the value in the 1-wk-old subject (*SI Appendix*, Fig. S22), which is related with spatiotemporal variation from the geometrical growth and organ development of the subject (33–35). Fig. 6*B* summarizes the heart and respiratory rate (26, 27) determined from such data averaged over a recording time of 60 min at each of these three ages in the form of bar graphs for LR (gray) and ER (red) subjects. Both subjects exhibit heart rates of 120 to 160 beats per minute and respiration rates of 38 to 45 RPM. These same CORB sensors also have temperature-sensing capabilities that can be used to determine not only estimates of core body temperature from the device on the chest but also peripheral temperatures as a measure of overall circulatory health (Fig. 6*C*).

## Discussion

The collection of soft, lightweight wireless sensors introduced here have potential as a widely deployable, significant improvement over standard clinical screening tools, with the possibility for routine automated, quantitative assessments in home/community settings for long-term monitoring. These technologies have designs optimized for use in children starting from very young newborn infants, with features that exploit infant-safe, low-cost components and high-volume manufacturing techniques. Such features, together with the intrinsic ease of use, facilitate access to healthcare professionals and parents alike. Recapitulation of 3D motion trajectories using remote computing resources provides a behavior visualization mechanism that can be assessed by clinicians across the world, without privacy concerns or geographic constraints. Outputs from these sensors may be further linked to established educational resources on motor development (e.g., ref. 23) via mobile applications to support early detection of abnormalities in the home and clinical settings. In the future, these foundational data may also serve as the basis for automated scoring of traditional GM assessments and other clinical tests using advanced machine learning techniques. Simultaneous capture of a broad range of vital signs information may provide additional important information, not only in the context of motion behaviors but in general health monitoring. Further potential is in the use of these devices to track physical rehabilitation therapies performed in the home or the clinic including strength training and postural stabilization (36, 37). Planning for a clinical trial that will test the utility of the sensors for efficient detection of neonatal motor atypicality as well as capacity for tracking neuromotor intervention effects is currently underway.

## Materials and Methods

### Procedures for Device Fabrication and Encapsulation.

Commercial software (AUTODESK EAGLE [version 9.6.0]) served as the basis for generating schematic diagrams and layouts for the FPCB. Customized firmware was downloaded to the BLE SoC using the Segger Embedded Studio. Placing components, soldering them to the FPCB, and folding the system completed the fabrication of the electronics. Aluminum molds prepared with a freeform prototyping machine (Roland MDX 540) defined the shapes of top and bottom layers of silicone (Silbione-4420, each 300 μm thick). Placing the electronics on the bottom layer while in its mold and pouring a precursor to a soft silicone elastomer (Eco-Flex 0030, 1:1 ratio) followed by mounting the top layer and clamping the system together prepared the system for curing the precursor at 95 °C in an oven for 20 min. After cooling to room temperature, removing the clamps and cutting excess material from the perimeter with a CO_2_ laser yielded the final device.

### Techniques for Modeling the Mechanical Characteristics.

The commercial software ABAQUS (ABAQUS Analysis User’s Manual 2010, version 6.10) was used to determine the strain ε distribution in the encapsulation and metal layers of the sensor under bending deformations. The soft encapsulated sensor, with an equivalent bending stiffness of approximately 3.4 Nmm^2^ (*SI Appendix*, Fig. S23), was modeled by tetrahedron elements (C3D10), and the thin Cu and PI films were modeled by composite shell elements (S4R). The number of elements in the model was ∼3 × 10^5^, and the minimal element size was one-eighth of the width of the narrowest interconnects (100 µm). Mesh convergence was guaranteed for all cases. The elastic modulus (E) and Poisson’s ratio (ν) are *E*_Ecoflex_ = 60 kPa and *ν*_*Ecoflex*_ = 0.5 for soft encapsulation, *E*_Cu_ = 119 GPa and *ν*_*Cu*_ = 0.34 for copper, and *E*_PI_ = 2.5 GPa and *ν*_*PI*_ = 0.34 for PI.

### Procedures for Full Body Motion Measurements.

The studies were approved by Northwestern University (STU00207900). For subjects, double-sided medical silicone adhesive (2477P; 3M) was applied to the device and secured the sensors to each limb. The legal guardians of the child provided informed, written consent for study images to be published with faces blurred.

### Protocols for Human Subject Studies.

The studies were approved by the Institutional Review Boards of Lurie Children’s Hospital (#2018-2098) and Northwestern University (STU00207900). For the LR and ER infants, devices were secured to the arms and legs using latex-free, self-adhering elastic bandage wraps (CoFlex; Andover Healthcare). The device on the chest was secured with hydrogel (Katecho, Inc.) The device on the forehead was held in place used a headband. The legal guardians of these infants provided informed, written consent for study images to be published with faces blurred.

## Data Availability

All study data are included in the article and/or supporting information. Additional supporting data are available from Zenodo (https://doi.org/10.5281/zenodo.3690141).
